# Intubation with Airtraq^™^ laryngoscope in a morbidly obese patient

**DOI:** 10.4103/1658-354X.76482

**Published:** 2011

**Authors:** Pratik Tantia, Sunny Malik, Shahin Jamil, Rajiv Samal

**Affiliations:** *Department of Anaesthesiology, J.N. Medical College Hospital, Aligarh Muslim University, Aligarh, India*

**Keywords:** *Airtraq^™^ optical laryngoscope*, *difficult intubation*, *morbidly obese*

## Abstract

In the present study, we report a case of successful endotracheal intubation using Airtraq^™^ Laryngoscope (AQL) in a morbidly obese patient. A 35-year-old woman, morbidly obese (weight, 105 kg; height, 160 cm; BMI, 41 kg/m^2^), known hypertensive and diabetic, was admitted in the operating room for total abdominal hysterectomy under general anesthesia. The preoperative airway assessment anticipated both difficult bag-mask ventilation and intubation. Tracheal intubation using AQL was attempted after induction with propofol and relaxation with succinylcholine. Successful tracheal intubation was accomplished within 12 seconds of insertion of AQL into the oral cavity. The minimal hemodynamic response during this maneuver was advantageous in our patient.

## INTRODUCTION

Morbidly obese patients (body mass index [BMI] > 40) (BMI = weight in kilogram/[height in centimeter]^2^) present a challenge to the anesthesiologist in terms of securing the airway. Predictors of difficult laryngoscopy and intubation in these patients include fat face and cheeks, large breasts in females, limited range of motion of head, neck and jaw, small mouth and a large tongue, excessive palatal and pharyngeal tissue, short thick neck (large circumference), high Mallampati scores (III or IV) and more rapid oxygen (O_2_) desaturation.[1] Associated comorbidities such as hypertension and diabetes add to the already difficult situation in terms of exaggerated hemodynamic response to laryngoscopy and intubation.

The Airtraq^™^ (Prodol Meditec S.A., Vizcaya, Spain) optical laryngoscope is a useful tracheal intubation device that can be used in patients with potential difficult airway, as in our case. It shortens the duration for tracheal intubation, and thus prevents reduction in arterial O_2_ saturation.[2] Because of less forceful blade elevation, maneuvering is gentle with minimal hemodynamic alterations,[3] adding to the advantage in hypertensive patients.

We report a case of successful and rapid (12 seconds) endotracheal intubation in a morbidly obese patient using AQL.

## CASE REPORT

A 35-year-old woman, with 105 kg weight and 160 cm height (BMI, 41 kg/m^2^), having dysfunctional uterine bleeding unresponsive to medical management, was admitted in the operating room for total abdominal hysterectomy under general anesthesia. She had well-controlled hypertension, diabetes mellitus type II, history of snoring and symptoms of gastroesophageal reflux. No history of obstructive sleep apnoea was detected. The preoperative airway assessment showed Mallampati class IV, interincisor gap of 3 cm, thyromental distance of 5.5 cm, and neck circumference of 55 cm. Head extension and cervical flexion were markedly limited. Patient was anemic with moderate pallor, resting pulse rate of 100/min, blood pressure of 150/94 mmHg, and fasting blood glucose of 116 mg/dl on the day of surgery, while other preoperative laboratory investigations were within normal limits. Informed consent was taken from the patient. Difficult airway cart was prepared, including the instruments of cricothyrotomy and tracheostomy, as difficulty in mask ventilation and intubation was anticipated. Endotracheal tube of 7.0 mm ID was lubricated and then passed through the side channel of Airtraq^™^ laryngoscope. Patient was put in supine position, her head supported with a soft pillow put under the head, and premedicated with Ondansetron 8 mg, Tramadol 200 mg and Midazolam 2 mg intravenously (i.v.). After preoxygenation with 100% O_2_ for 3 minutes, induction with propofol 200 mg i.v. and relaxation with succinylcholine150 mg i.v., endotracheal intubation was attempted with Airtraq^™^ laryngoscope. A full view of centered glottis could be obtained after elevation of the epiglottis and endotracheal tube could be passed through the vocal cords easily. Time taken from insertion of Airtraq^™^ into the oral cavity to successful endotracheal intubation was 12 seconds. Endotracheal intubation was confirmed by capnography and auscultation of the chest. During this time, patient’s pulse rate, blood pressure and ECG were within normal limits. No desaturation and mucosal bleeding occurred during intubation. Endotracheal tube was secured in place and the surgeons were asked to proceed with the surgery. General anesthesia was maintained with propofol infusion and mixture of O_2_ and nitrous oxide and neuromuscular blockade using vecuronium. The procedure lasted 2 hours; residual neuromuscular paralysis was reversed with neostigmine and glycopyrrolate, after which she was successfully extubated with an uneventful recovery. Patient maintained saturation in head-up position in the post-operative period on O_2_ through mask. The patient exhibited only mild symptoms of sore throat that improved within 2 days without any intervention; otherwise, the post-operative period was uneventful. No hoarseness and other airway complications were noted.

## DISCUSSION

Obesity can be a potential cause of complications after induction of anesthesia resulting from rapid desaturation,[4] limited mobility of atlanto-occipital and temporomandibular joints, narrowed upper airway, shortened distance between the mandibular and sternal fat pads, and delayed gastric emptying with gastro-esophageal reflux leading to pulmonary aspiration.[5]

AQL is a new device providing direct view of the glottis, with minimal maneuvering of patient for optimal positioning. There are reports demonstrating successful intubation using AQL in normal airways,[6] simulated difficult airways[7] and in a clinically difficult airway.[8,9] It has been used electively[10] as well as a rescue device[11] after failed attempt with conventional laryngoscopy.

Our patient, a morbidly obese woman (BMI, 41 kg/m^2^) was successfully intubated with AQL in 12 seconds. Ndoko *et al*. (2008) also demonstrated that AQL shortened the duration of tracheal intubation and prevented reduction in O_2_ saturation.

Hemodynamic changes (heart rate, blood pressure) were minimal during intubation using AQL. Similar findings have also been shown by Maharaj *et al*. (2007) and Ndoko *et al*. (2008).
